# Variables Associated With Successful Treatment Outcomes of Autistic Youth Enrolled in PEERS

**DOI:** 10.3389/fpsyt.2022.834801

**Published:** 2022-03-21

**Authors:** Justin W. H. McLeod, Adam W. McCrimmon

**Affiliations:** ^1^Werklund School of Education, University of Calgary, Calgary, AB, Canada; ^2^Alberta Children's Hospital Research Institute, University of Calgary, Calgary, AB, Canada

**Keywords:** autism, social intervention, outcomes, prediction, cognitive intelligence

## Abstract

This study sought to examine how certain variables of autistic youth who completed a formal social intervention program (PEERS) predicted social skill improvement post intervention. Specifically, this research aimed to determine if age, gender, emotional intelligence, intellectual ability, and/or autism symptomatology predicted social skill outcomes. Using extant data from parent and self-report batteries, change scores and multiple regressions were employed to examine which variables accounted for social skill improvement. Only intellectual ability (FSIQ), specifically perceptual reasoning, significantly predicted social skill outcomes based upon teen self-report, suggesting that autistic youth with specific cognitive profiles may be benefit more from PEERS. This research also exemplifies the heterogeneous nature of autism symptomology and the continued need for research examining social skill interventions. Limitations and future directions are discussed.

## Introduction

Social skills are essential for meaningful peer relations, prosocial behaviors, and developing positive connections with individuals in various settings (1, 2). Social skills are also associated with academic achievement, psychological adjustment, coping, and employment outcomes (3). The development of social skills follows various milestones from early infancy to adolescence, finally maturing but ever evolving in adulthood, allowing individuals to interact with their immediate social environment and develop the necessary abilities to engage in conflict resolution, reflective conversations, development of meaningful friendships, social perspective taking, and collaboration with others (4–7). However, underdeveloped social skills are a common occurrence and are associated with peer rejection and isolation, internalizing and externalizing disorders, peer victimization, academic and employment challenges, and a negative self-concept (8–10). Specific to autistic individuals, social skill challenges often encompass a lack of close peer and school staff relationships, peer victimization, and the development of varying mental health disorders. Indeed, autistic children appear to be victimized at a much higher rate than TD peers and may develop externalizing and internalizing disorders directly related to their social skill impairments (11, 12). It is apparent that effective and well-developed social skills are imperative for adequate academic, employment, and quality of life outcomes; however, social skills impairments and maladaptive social behaviors are an integral part of the diagnostic criteria and a main impairment observed in autism^1^.

As social skills may not develop naturally in autistic individuals, interventions that focus on social skill development are considered essential (15). Group social skills interventions (GSSIs) are often employed to improve autistic individuals' social skills. Of the GSSIs noted to be effective, The Program for the Enrichment and Education of Relational Skills [PEERS; (16)] emerges as an effective and highly researched program for autistic youth. Through cognitive behavioral therapy (CBT) techniques and co-occurring parent education sessions, PEERS improves social skill outcomes in both short- and long-term observations [e.g., (17)]. Specifically, PEERS improves participants' social skills knowledge, social responsiveness, and social cognition while simultaneously reducing autism symptomology (18, 19).

With PEERS demonstrating both efficacious and effective results for the improvement of social skills and reduction of autism symptomology, it becomes important to understand the potential predictors of social skill improvement in this intervention. Notably, little is known regarding the specific cognitive profiles, personal characteristics, and/or abilities that autistic individuals possess that may predict social skill improvement in PEERS. These predictors are important to consider as GSSIs could be produced and specifically aimed at individuals who will succeed, and novel intervention approaches could be created for those who do not experience treatment gains with current programs. The present study examined specific predictors that autistic individuals possess that may lead to improved social skills from PEERS to redress this gap within the literature.

## Social Skills

Social skills represent a dynamic interplay of genetics, ecological systems, and the individual themself, as these skills are mainly developed from childhood throughout adolescence and refined in adulthood, setting the stage for peer interactions and connection throughout an individual's life (20). Typically developing social skills, surrounding positive connections and acceptance, prosocial behaviors, and developing meaningful relations, are associated with beneficial psychosocial health and academic achievement (1, 2). Conversely, impairments to social skills may create current or future concerns regarding psychological distress, social isolation, and reduced self-esteem, all of which may greatly reduce an individual's quality of life (20). Importantly, as described by Gresham (1), the distinction between social skills, social competence, and social tasks must be made when conceptualizing social behavior. A social task may be interacting in a game with peers, having a phone call with others, or joining into a conversation. Social skills are the necessary characteristics and actions an individual exhibits to complete a social task, whereas social competence is the judgements of others on how the social task was completed. Therefore, according to Gresham et al. (21), social skills comprise specific behaviors exhibited to complete a social task that are then judged by external agents as either competent or incompetent. While it is important to examine social behavior, the present study and the measures employed specifically examine social skills and the improvements observed through social (skill) interventions.

## Autism

Autism was first introduced in the early twentieth century; however, its conceptualization has evolved as research and understanding of it has developed. Currently, autism is a neurodevelopmental disorder in the *Diagnostic Statistical Manual of Mental Disorders* [5th ed.; *DSM-5*; (22)] characterized by impairments in social and communicative abilities in conjunction with restricted and/or repetitive interests and behaviors (RRBs). Communication and social skills prove challenging for autistic individuals given the required impairments in language development, poor non-verbal skills, and issues reading social cues inherent to the diagnosis (23). The RRBs and social impairments typically present in early childhood yet are variable and may evolve throughout an individual's lifespan (22). While there have been changes in the way researchers operationalize autism, the core features have remained relatively unchanged (23). However, autism has recently subsumed other historical diagnoses, resulting in it being regarded as a spectrum with severity of symptoms ranging from mild to severe.

## Social Skills and Autism

Autistic children demonstrate impairments in various social skill domains when compared to typically developing (TD) peers (24). Domains such as self-control, cooperation, and assertion, all of which are pertinent for social success, are less developed in autistic children compared to TD controls (24). Social skill impairments affect basic social interactions and the development of social relationships (25). These impairments affect autistic children at all ages, as an individual moves through various stages from imaginative play as a child to establishing close personal friendships in adolescence. Social skill impairments encompassing challenges with communication and lack of reciprocal friendships are directly associated with peer rejection, poor peer relationships, and inferior quality relationships with school staff (26, 27). These challenges surrounding the development of reciprocal friendships and associated peer rejection relate to autistic youth becoming the targets of bullying, victimization, and isolation (28). Regrettably, the lack of ability to develop close friendships or being the victim of peer rejection has monumental effects as almost half of autistic youth have at one point had suicidal ideation or attempted suicide (12).

## Emotional Intelligence and Autism

EI, which is necessary for processing emotional information and utilizing it to solve problems and navigate social situations, is underdeveloped in many autistic individuals (29). In fact, many autistic individuals have difficulties understanding complex feelings and emotions, making socially related inferences, and managing their emotions appropriately (30, 31). With a core feature of autism being impairment in social communication and interaction (22), it is no surprise that many autistic individuals demonstrate challenges with EI. Indeed, social interactions require individuals to recognize one's own emotional state and regulate one's emotions to respond appropriately, which is epitomized by both social skills and EI (32). In general, low levels of EI relate to challenges with engagement in social interactions, regulation of emotions, and processing of external emotional information, leading to poor reciprocal friendships and negative peer interactions (33, 34).

## Intelligence and Social Skills

Research into cognitive profiles and social skills is limited. Individuals with high verbal ability and relatively lower non-verbal ability often present with social skill difficulties [e.g., (35, 36)]. As described by Kimpton (35), individuals with this cognitive profile are often diagnosed with a non-verbal learning disability and experience higher rates of social skill difficulties (35). This cognitive profile also has implications for the autistic community as autistic individuals with higher verbal ability demonstrate lower levels of autistic symptoms (37) and those with lower verbal and higher non-verbal ability demonstrate increased autism symptoms (38). Despite these findings, the complex interplay between intelligence and social skills is largely unknown.

## Age and Gender Differences In Social Skills

Both age and gender influence social skills. Regarding age, it is apparent that over time neural, cognitive, and behavioral mechanisms such as sensory, motor, language, and working memory ability allow individuals to manage their social environments appropriately (6). Additionally, age affects social responses to varying situations. Most notably, as age increases so does the ability to engage in peer interactions effectively and social problem-solving strategies (39). With respect to gender and its influence on social skills, males and females respond to and think about social situations in contrasting ways [e.g., (40)]. In fact, Walker et al. (39) observed that increased prosocial responses were noted during female social interactions whereas increased aggressive or retaliatory actions were noted in males. While these findings related to gender and age do not necessarily predict social skill success, they demonstrate the unique influences that both variables have on social interactions.

## Peers

PEERS is an evidenced based social intervention aimed at teaching necessary skills for making and keeping friends, managing peer conflict, and addressing peer rejection to autistic teens (16). PEERS is designed for teens (13–18 years of age) experiencing challenges with a broad range of social skills (16). A main tenet of PEERS is that the intervention is parent-assisted, allowing parents to learn the taught skills and to act as social coaches for their teen. PEERS consists of 14 sessions of 90 min each that cover various social skills such as electronic communication, having get-togethers, and handling teasing and/or bullying (16). Unique to PEERS is the focus on homework assignments, parent handouts, role-plays, behavioral rehearsals, and an emphasis on teens engaging in get-togethers with peers (16). The techniques specific to PEERS surround the use of CBT in the form of didactic instruction, Socratic questioning, perspective taking, behavior rehearsal, homework assignments, and social problem solving that encourage teens' engagement within the group and increases the durability of treatment gains over time (17).

Numerous RCTs have evaluated the efficacy of PEERS at reducing autism symptomology and improving social skills in adolescents [e.g., (41)]. Both short- and long-term gains have been found; however, predictors of this success as well as the reasons for a lack of treatment gains is understudied (42, 43). Chang et al. (42) determined that adolescents with higher parent-reported baseline social skills demonstrated greater improvement in social skills following PEERS. Regarding gender, Mcvey et al. (43) concluded that there were no gender differences in the outcome of PEERS as both male and female participants responded similarly to the intervention. However, Chang et al. solely relied on parent reported social skill improvements, used a small number of predictor variables, and did not report reliable change scores. Additionally, Mcvey et al. (43) studied only the effects of gender on post-PEERS social skill gains. Nonetheless, this research is important as predictors of success in GSSIs are not understood; such knowledge would clarify factors that may lead to social skills improvements in PEERS and other GSSIs.

## Current Study

While GSSIs, and specifically PEERS, have been extensively studied, not every participant demonstrates the social skills gains from the intervention largely reported in the literature [e.g., (44)]. In fact, many teens do not improve in the targeted skills and some even regress. We do not yet understand which individuals and what characteristics they possess that may lead to social skill improvement within PEERS. To redress this gap within the literature, autism symptomatology, EI, cognitive intelligence, gender, and age were examined to understand which predict social skill change in PEERS. Specifically, this project sought to answer the question of which of the variable(s) included in the analysis may significantly predict social skills changes upon completion of PEERS.

## Participants

Extant data from 65 participants were analyzed to examine specific variables' prediction of improvement of social skills from completion of PEERS. The sample included 58 males (89%) and 7 females (11%), with an average age of 15.66 years (SD = 1.48; range = 13.30–18.80). The data was collected and adhered to all informed consent and ethical/regulatory guidelines. All participants were required to undergo a screening process, including a parent and child interview, formal evaluation of behaviors related to autism, as well as an online questionnaire to assess eligibility for PEERS and to acquire relevant background information. The extant data were also required to include pre and post social skills data, and data on cognitive intelligence, EI, and autism symptomatology. Demographic information is shown in Table 1.

**Table 1:** Demographic information.

	**Mean (SD)**	**Minimum**	**Maximum**
Age (years)	15.66 (1.48)	13.30	18.80
Gender (% male)	89.23		
FSIQ-4	104.91(15.34)	70.00	130.00
PRI	106.26 (17.78)	71.00	147.00
VCI	102.34 (14.81)	73.00	137.00
Change score parent	5.33 (9.40)	−14.00	34.00
Change score teen	4.83 (8.91)	−14.00	22.00

*Age is presented in decimal form (i.e., 15 years, 6 months = 15.5). FSIQ is presented in standard score format (M = 100, SD = 15). Change scores reflect the difference in SSIS standard scores from pre- to post-test*.

## Measures

### Autism Diagnostic Observation Schedule, Second Edition

The ADOS-2 (45) is a standardized measure of autistic symptomology; particularly social affect and RRBs. The ADOS-2 has five separate modules, one of which is selected for completion based on an examinee's language ability and chronological age. In the present study, all participants completed either module three (under 16 years of age) or four (16 years or older). Internal consistencies are adequate for both the SA domain (0.84) and for the RRB domain (0.61), respectively. All administrations for the present research were administered by reliable clinicians and were recorded and reviewed by a certified ADOS-2 trainer to ensure administration and results. Participants were required to exceed the algorithmic threshold indicative of a diagnosis of autism.

### Social Skills Improvement System

The SSIS (46) measures social skills of 8- to 18-year-olds. The SSIS is concerned with the frequency and perceived importance of positive behaviors demonstrated by the student as well as problem behaviors that impact a student's ability to engage in appropriate social skills (46). Internal consistency levels are 0.90 or higher with median alpha values of the subscales ranging from 0.70 to 0.80 on parent/teacher forms and self-report forms, respectively. Only the social skill domain was used in the current study, consisting of areas such as communication, cooperation, assertion, responsibility, empathy, engagement, and self-control. Reliability and validity are deemed adequate, with high alpha values observed regarding reliability and factor analysis demonstrating strong validity of the measure [see Crosby (47)].

### Wechsler Abbreviated Scale of Intelligence, Second Edition

The WASI-II (48) is an abbreviated measure of cognitive ability designed for individuals between 6 and 90 years of age. The WASI-II's technical adequacy is strong, with satisfactory test standardization, reliability, and validity (49). In terms of the WASI-II's reliability, internal consistency scores of the child sample ranged from good (0.87) to excellent (0.91), with the adult sample demonstrating excellent consistency scores (0.90–0.92). For this study, the four-subtest form was employed to garner verbal comprehension (VCI), perceptual reasoning (PRI), and full scale (FSIQ) scores to be used in the analysis. Participants were required to demonstrate VCI, PRI, and FSIQ above 70 to establish ability to complete the self-report questionnaires and in alignment with the recommendations of the developers of PEERS that those completing the program not present with intellectual impairment. While the four-subtest form was used to glean verbal and perceptual reasoning abilities, only PRI and FSIQ were included in the analysis as non-verbal reasoning is directly related to social skill acquisition in autistic individuals, whereas verbal reasoning is more related to receptive and expressive language abilities and not real-world social skill ability (50). Moreover, including VCI in the analysis may have created multicollinearity issues or led to the model being overfit, producing poor predictions on social skill improvement. Therefore, VCI was used for screening participant cognitive ability but was not included in the predictive model.

### BarOn Emotional Quotient Inventory: Youth Version Short Form

The BarOn EQ-I: YV(S) (51) is a measure of EI such as one's ability to understand feelings, empathize with others, and adapt to novel social situations. The BarOn EQ-i:YV (S) demonstrates adequate technical quality in regard to the normative sample, reliability, and validity. Internal consistency reliability estimates ranged from 0.65 to 0.87. For this study, only the total EI and intrapersonal scale scores were analyzed as the total EI score provides a holistic examination of an individual's EI and the intrapersonal subscale examines constructs explicitly related to social skills and areas targeted by PEERS. The intrapersonal subscale is the most comprehensive, exploring five domains necessary for social competence (e.g., self-regard, emotional self-awareness, assertiveness, independence, and self-actualization). As outlined by Wood et al. (52), the intrapersonal subscale embodies the ability to identify, label, and understand emotions, which are necessary for developing appropriate social behaviors through understanding other social agents' perspectives and emotional responses. Additionally, PEERS specifically targets participants' ability to take the perspective of others, which is necessary for informing social behaviors, such as if someone is interested in a conversation you may be engaging in with them.

### Social Responsiveness Scale–Second Edition

The SRS-2 (53) is a rating scale concerned with social behavior and communication impairments commonly related to autistic symptomology. The SRS-2 demonstrates excellent reliability and validity on measures examining technical adequacy (54). The internal consistency of the SRS-2 is strong with reliability coefficients ranging from 0.94 to 0.96 across all age bands in both a clinical and normative sample. Of the 65 items, results are reported in subscales (Social Awareness, Social Cognition, Social Communication, Social Motivation, and Restricted Interests and Repetitive Behavior) that cover social skills and common autistic symptomology as well as a total overall score; only the total overall score of the five subscales was used in the analysis. This decision reflects the desire to observe how overall social communication impairments related to autism symptomatology may predict social skill improvement post PEERS. Since all five subscales are associated with autistic symptomology, it proved superfluous to examine any subscales individually.

## Procedure

### Data Collection

Extant data from a 6-year period, ranging from 2013 to 2019, was utilized for this study. Only data pertaining to social skill, cognitive, autism symptomatology, and EI were included. Social skill data was examined through change score analysis; data gathered at two time points were used for this purpose: 1 week before (T1) and 1 week after (T2) the intervention.

During the period in which data was collected, PEERS research and the PEERS intervention were distinct entities. Families who were accepted into PEERS were provided with the choice to participate in research or solely engage in the intervention. Teens who accepted research participation invites completed various measures (e.g., ADOS-2, WASI-II) to ensure they met inclusion criteria. Individuals who fell below eligibility criteria (i.e., failed to exceed threshold for a diagnosis on the ADOS-2 and/or scored below 70 on either VCI or PRI components of the WASI-II) were able to complete the intervention but were not included in the research. Participating parents and teens were provided monetary compensation for their time in completing the measures.

### Intervention

Eligible teens and their parent(s) participated in PEERS facilitated by registered psychologists and graduate student clinicians who were certified in the program's delivery. The intervention team consisted of two PEERS facilitators (one for the teen group and another for the parent group) and two behavioral coaches (both in the teen group) who supported the teen facilitator via demonstrative role-plays, assisting in behavioral rehearsals, and promoting appropriate behavior of participants. Participants from 13 cohorts a included in this study.

## Analysis

SSIS ratings for both pre- and post-intervention were completed separately by teens and parents to derive a change score. Change scores provide a metric of the raw gain observed by individuals as an index of change over time or the difference between two measures using the same sampling unit (55). By comparing pre-post change, the statistic examines the null hypothesis of no difference across measurements in the amount of raw change between the time points (56). Quite simply, a positive value signifies an observable amount of positive change or a successful intervention. Conversely, a negative value indicates an observable amount of declining skill over the two time periods. Finally, no difference (a change score of zero) indicates no change of skill or behavior. While the methodology of measuring growth and change through two time points has been used in psychological research for years, many studies use the analytical method incorrectly [(57); see also (55, 58, 59)].

The present analysis ensured reliable change scores and followed procedures to avoid fallacious conclusions and avoiding measuring artifacts of the statistical method to combat the possible flaws associated with change scores. It was imperative that the change score measure be reliable to determine if change, such as social skill improvement, is measured accurately. Three sets of information were used to determine the reliability of two (parent SSIS and student SSIS) change scores used in this study. The first value used in the reliability calculation is the estimated reliability of each of the two tests used to compute the difference score (i.e., pre- and post-test SSIS). Second, the variability of the tests' observed scores must be examined. Finally, the correlation between observed test scores is calculated. Once those values are obtained, they are placed in the following equation:


$$\begin{array}{l} R_{d}=\;\frac{s^{2}x_{0}R_{xx}+s^{2}y_{0}R_{yy}-{2r}_{x_{0}y_{0}}s_{x_{0}}s_{y_{0}}}{s^{2}x_{0}+s^{2}y_{0}-\;{2r}_{x_{0}y_{0}}s_{x_{0}}s_{y_{0}}}\; \end{array}$$

The change scores in the present study yielded reliability coefficients of 0.80 and 0.79 for parent and student change scores, respectively. Parent change score reliability alpha (0.80) represents a good range, whereas student change score reliability alpha (0.79) represents an acceptable range according to Cronbach and Furby (59). While there are varying reports on reliability ranges, it is largely agreed that reliability coefficients must be above 0.70 to be regarded as acceptable, and usually range from 0.70 to 0.95 (60, 61).

As two change scores were used in the analysis, the research questions examining influence of intellectual ability, EI, autism symptomatology, age, and gender on the outcome of social skill improvement through PEERS were examined through two lenses: parent and self-report. This is important because teens and parents may rate social skill improvement differently both before and after they have completed PEERS. Two multiple regressions were used for parent and teen change scores to observe which variables affect variation in social skills post intervention. Multiple regression allows this study to determine the overall model fit (variance explained) and the relative contribution of each predictor [age, gender, autism symptomatology (total SRS-2 score), EI (BarOn EQ-I: YV(S) intrapersonal subscale and total EI score), and intellectual ability (WASI-II FSIQ and WASI-II PRI)] to the total variance explained in social skills change scores.

## Results

### Teen Change Score

A multiple regression was run to predict social skill improvement from age, gender, autism symptomatology (total SRS-2 score), EI (BarOn EQ-I: YV(S) intrapersonal subscale and total EI score), and intellectual ability (WASI-II FSIQ and WASI-II PRI). After the data was cleaned, there was linearity as assessed by partial regression plots and a plot of studentized residuals against the predicted values. There was independence of residuals, as assessed by a Durbin-Watson statistic of 2.075. There was homoscedasticity, as assessed by visual inspection of a plot of studentized residuals vs. unstandardized predicted values. There was no evidence of multicollinearity, as assessed by tolerance values >0.1. There were no studentized deleted residuals >±3 standard deviations, no leverage values >0.2, and values for Cook's distance above 1. The assumption of normality was met, as assessed by a Q–Q plot and histogram. The results of the regression indicated that the model explained 34.3% of the variance. The multiple regression model was statistically significant in predicting teen social skill change scores, *F*_(7, 52)_ = 3.833, *p* < 0.05, adj. *R*^2^ = 0.26. However, only the WASI-II FSIQ-4 (*B* = 0.49, *p* < 0.001) and WASI-II PRI (*B* = −0.49, *p* < 0.001) variables added statistical significance to the prediction. The size and direction of the relations suggest that higher overall FSIQ with lower PRI predicts improved social skills and, in turn, better outcomes from PEERS. Results of this analysis can be seen in Table 2.

**Table 2:** Multiple regression results for teen change scores.

**Change Score**	***B***	**95% CI for *B* LL**	**95% CI for *B* UL**	***SE B***	**β**	***R^**2**^***	**R^2^**
Model						0.59^*^	0.34^*^
Constant	−7.655	−47.073	31.762	19.643			
Gender	−1.912	−9.041	5.217	3.553	−0.064		
Age	0.785	−0.736	2.305	0.758	0.124		
WASI-II FSIQ-4	0.488^**^	0.248	0.729	0.120	0.825^**^		
WASI-II PRI	−0.494^**^	−0.691	−0.296	0.098	−0.975^**^		
Baron intra	0.081	−0.113	0.275	0.097	0.142		
SRS-2	−0.057	−0.301	0.187	0.122	−0.055		
Baron TEQ	0.006	−0.186	0.198	0.096	0.009		

*Mode, “Enter” method in SPSS statistics; B, unstandardized regression coefficient; CI, confidence intervals; LL, lower limit; UL, upper limit; SE B, standard error of the coefficient; β, standardized coefficient; R^2^, coefficient of determination; ΔR^2^, adjusted R^2^*.

* *p < 0.05*,

** *p < 0.01*.

### Parent Change Score

A multiple regression was run to predict social skill improvement from age, gender, autism symptomatology (total SRS-2 score), EI (BarOn EQ-I: YV(S) intrapersonal subscale and total EI score), and intellectual ability (WASI-II FSIQ and WASI-II PRI). After the data was cleaned, there was linearity as assessed by partial regression plots and a plot of studentized residuals against the predicted values. There was independence of residuals, as assessed by a Durbin-Watson statistic of 2.044. There was homoscedasticity, as assessed by visual inspection of a plot of studentized residuals vs. unstandardized predicted values. There was no evidence of multicollinearity, as assessed by tolerance values >0.1. There was one case of studentized deleted residuals >±3 standard deviations; however, it was not deemed to be an influential data point. There were no leverage values >0.2, or values for Cook's distance above 1. The assumption of normality was met, as assessed by a Q-Q plot and histogram. The results of the regression indicated that the model explained 6.1% of the variance. The multiple regression model was not statistically significant in predicting parent social skill change scores, *F*_(7, 49)_ = 0.457, *p* = 0.86, adj. *R*^2^ = −0.07. Moreover, none of the variables significantly added to the prediction. Results of this analysis can be seen in Table 3.

**Table 3:** Multiple regression results for parent change scores.

**Change score**	***B***	**95% CI for *B* LL**	**95% CI for *B* UL**	***SE B***	**β**	***R^**2**^***	**R^2^**
Model						0.06	−0.07
Constant	−10.078	−57.327	37.172	23.512			
Gender	−3.477	−11.604	4.650	4.044	−0.127		
Age	0.781	−1.036	2.597	0.904	0.127		
WASI-II FSIQ-4	−0.149	−0.446	0.148	0.148	−0.247		
WASI-II PRI	0.139	−0.114	0.392	0.126	0.267		
Baron intra	−0.026	−0.270	0.218	0.121	−0.044		
SRS-2	0.150	−0.167	0.466	0.157	0.140		
Baron TEQ	−0.007	−0.255	0.240	0.123	−0.012		

*Mode, “Enter” method in SPSS statistics; B, unstandardized regression coefficient; CI, confidence intervals; LL, lower limit; UL, upper limit; SE B, standard error of the coefficient; β, standardized coefficient; R^2^, coefficient of determination; ΔR^2^, adjusted R^2^*.

## Discussion

The first variable examined if age would relate to varying outcomes from completion of PEERS. Previous research on the effects of baseline age in predicting positive social skills in PEERS indicated no effect (42). Analysis of parent and teen change scores through respective multiple regressions revealed that neither participant group reported a significant effect of age (*p* > 0.05). The lack of significance of age in the current study could be because other variables that are not affected by age may play a larger role in social skill improvement. For example, participants may have varying levels of intelligence, social awareness, or participation levels, all of which may have more greatly affected social skill improvement regardless of age. Moreover, PEERS is designed to be administered to individuals aged 13–18, with no known age differences in terms of the digestibility of content. Therefore, the result observed in this present study with age not being a significant predictor of social skill improvement further proves that the age ranges for the PEERS program are appropriate, with all participants having equal opportunity to succeed.

The second variable examined if participant gender would predict the outcome from the completion of PEERS. Past examination of participant gender's possible influence on social skill improvement through PEERS was examined by Mcvey et al. (43), with no significant effect being observed. Indeed, the same was observed in this study with there being no significant prediction of social skill improvement in PEERS based on gender (*p* > 0.05). Like the variable of age, gender simply may not create advantages or disadvantages for autistic individuals in terms of opportunity to improve their social skills. Instead, other characteristics that autistic individuals possess may be of more consequence to learning and maintaining new social skills. Interestingly, there has been research illustrating that autistic females may have social advantages due to the ability to mask autism symptoms (62), mimic others' social behavior better than males (63), and score significantly higher on friendship quality measures (64). Conversely, it has been found that autistic females may be equally or more socially disadvantaged than males (65). The divergent findings of certain social advantages or disadvantages that autistic females may have, combined with the findings of the present study, demonstrating no significant effect of gender on social skill improvement embodies the heterogeneous nature of autism symptomology. Overall, through conflicting previous research findings and the current study, it appears as though gender is not a main predictor of the ability to learn and progress social skills in PEERS.

The third variable of examining participant intellectual ability and EI forecasting social skill outcomes post completion of PEERS demonstrated mixed results. There is no known research examining the relation between emotional and cognitive intelligence with social skill improvement through PEERS. Nonetheless, research has demonstrated the importance of EI in overall social functioning and social relationships [e.g., (66)]. It is also established that lower cognitive ability in some autistic individuals is associated with greater social skill challenges (67). While EI is important for social relationships and developing adequate social skills, the present study did not find that EI predicted better social skill outcomes.

Cognitive ability predicted social skill gains; teens' higher FSIQ predicted social skill improvement (*p* < 0.001). Interestingly, a lower PRI score also predicted social skill improvement (*p* < 0.001), meaning that a profile of a high VCI that offset a poorer PRI to yield an overall strong FSIQ predicted social skill improvement. Specific to autism, it has been recently observed that autistic individuals with higher verbal ability demonstrate lower levels of autistic symptoms (37). Consequently, reduced autism symptoms observed in cognitive profiles with higher VCI may explain the present findings.

Previous research along with the present findings may lend weight to the idea that individuals with a higher FSIQ and VCI but lower PRI may understand the material presented to them better and be able to apply the learned social skills due to higher verbal ability and better rote memory of certain social scenarios and applicable rules. Conversely, individuals with high VCI and low PRI may have more room to grow with social skills due to their lack of non-verbal knowledge. An example of PRI skills in PEERS is the ability to assess for interest of others during a conversation. Specifically, participants learn to examine the body language of another individual such as if they are making eye contact with them or facing them to understand if the person is interested in the conversation. Therefore, an individual who may struggle with utilizing their visual-spatial and novel problem-solving skills may have a higher trajectory of learning non-verbal cues, further influencing their overall social skill improvement in PEERS. While PEERS focuses on numerous social skills, the program may pinpoint PRI/non-verbal social skill development such as non-verbal cues that may be lacking in some individuals that in turn are useful in the enhancement of social skills. An additional explanation may have to do with VCI's association with reduced autism symptoms. Particularly, individuals with a higher VCI, and therefore lower autism symptomology, may be better able to overcome EI and/or social cognitive impairments that forecast social skill challenges. Conversely, autistic individuals with lower verbal abilities, and therefore increased autism symptomology, may have challenges reasoning with PEERS information due to more impacted EI and social cognitive abilities. Whether it is an ability to understand, memorize, and utilize learned social skills or having increased room to grow with non-verbal social skills, the present finding illustrates that a higher overall FSIQ and VCI while having a lower PRI score leads to predicting social skill improvement in PEERS.

Although cognitive ability appears to play a role in self-report predictors of social skill change, the same was not upheld in the parent report measure. This disparity between parent and self-report may demonstrate how parents view their adolescents' social skills differently and how variables may affect perceived social skill improvement uniquely.

The final variable examined was autism symptomatology and how it may relate to the participant social skill outcome. Current understandings of autism symptomatology illustrate that comprehending and predicting others' social behaviors and mental states may be a challenge for many autistic individuals [e.g., (68)]. Specifically, emotion recognition, empathetic ability, and dyadic/triadic interactions appear to be lagging skills for many autistic individuals, leading to challenges with social interactions. Interestingly, results revealed that autism symptomatology did not predict social skill outcome (*p* > 0.05). With this variable playing an imperative role in social interactions, it is puzzling that this factor was not influential in determining social skills post intervention. However, this finding may be explained by the intervention's focus on social skill development that does not necessarily impact upon autism symptomatology. Indeed, PEERS is not designed to address behaviors of autism and, while autism symptomatology is important for predicting and reacting to others' emotional or mental states, PEERS solely attends to developing the social skills to make and keep friends, and function appropriately in varying social situations. Additionally, as autistic individuals present with specified behavioral indicators of the diagnosis, there may have not been enough variance between participants' SRS-2 scores to observe a significant difference.

## Implications

The findings of the present study, while exploratory in nature, carry important implications for both the research and field of practice regarding social skills, autism symptomology, and predicting successful outcomes of PEERS. Most notably, recruiting participants who have a cognitive profile of higher FSIQ and VCI, with lower PRI, may lead to significantly improved social skill outcomes in PEERS. While PEERS is effective and highly supported by research leading to social skill improvement for many autistic individuals, it may prove more beneficial for individuals demonstrating social skill challenges with the aforementioned cognitive profile. No matter the root of the predicted social skill improvement due to specific cognitive profiles, the ability to significantly improve self-reported social skills should be fostered and therefore pre-screening may be effective in finding individuals who may have the most treatment gains.

## Limitations

While the findings of this study do carry important implications, there are many limitations; interpretation of the results should be done with the following in mind. A major limitation of the study surrounds various issues regarding the sample of extant data and participants included in the analysis. The sample size was relatively small, which reduces the generalizability of the findings. As well, nesting effects may have been present due to the study including various cohorts. Moreover, although this study was aimed at examining social skill outcomes of autistic teens post PEERS intervention, it may not be representative of a randomly selected sample of autistic teens. In general, the extant data represents autistic teens who were willing to engage in a GSSI and its associated programming. Additionally, participants were selected with strict inclusionary measures; this sample does not represent the true heterogeneity of autistic individuals such as individuals with intellectual disabilities. Finally, the sample lacked equal group sizes specific to female participants. Although it is well-established that there is a high male to female ratio in autism (69), it would prove beneficial to have a larger proportion of female participants to understand gender differences better.

The disparity between variance of the self-report and parent report change score models is also a limitation of the present research. Likely due to parents only observing their child in a restricted environment or their children attempting to strive for autonomy and distancing themselves from social interactions with parents, this disparity may have been satisfied with a third agent report. Specifically, this study lacked the insight from teachers, which could have furthered the insight into social skill improvement post PEERS in an environment where teens most often engage their social skills with peers and individuals in authority. Teacher reports were administered as part of the initial data collection; however, most respondents failed to return the reports or did not fill them out correctly. As such, this study lacks the insight of specific third parties and reduces the insight into predictors of social skill change post PEERS.

Building upon the limitations regarding the rating methods of social skill improvement, there is the issue of self-report biases. Specific to autistic teens rating their social-emotional functioning, many researchers have found significant discrepancies between self-report and parent measures [e.g., (70)]. It is possible that the self-reports utilized in the present study fell victim to selective memory biases, such as teens only recalling positive or negative social interactions, instead of viewing their pre and post ratings in a holistic sense. Additionally, many participants may have initially rated their social skills highly and then realized that they may have overrated themselves and in turn rated themselves lower on post report measures when observing other participants and learning novel social skills through PEERS. The opposite may have also been true, with participants viewing their self-efficacy and social skills lower than they may have been and then reporting larger gains than what were really occurring. Due to the possible discrepancies and concrete observations of possible self-report biases in autistic populations, self-report measures must be interpreted with caution.

Indeed, while GSSIs are aimed at improving social skills for autistic youth, there is discussion surrounding whether the interventions improve social skills or teach participants ways to compensate or camouflage social skill deficits and autism symptoms. Supporting this notion, research has demonstrated that in many autistic individuals, overt behavior or perceived ability of social skills is substantially better than their measured ability on social cognition, EI, or intelligence measures (71). Challenges with EI and social cognitive processes spur difficulties with emotion regulation, reading others mental states, and reasoning with emotional stimuli and are well-documented associated variables for social skill deficits [e.g., (72)]. If participants demonstrate continued challenges with social cognitive or EI abilities, it may indicate that participants have only learned skills to compensate or camouflage their social skill challenges. Unfortunately, Livingston (71) reports that many of these compensatory strategies are fragile and can be easily overwhelmed in challenging or evolving social situations. Since there is disparate evidence that GSSIs may only provide fragile skills to compensate for social skill difficulties, there is limitations to this studies ability to make generalizable conclusions to social skill improvement.

Although this study attempted to measure variables predictive of social skill outcomes, there is the possibility that other variables could better predict social skill outcomes. While it is challenging to measure every variable or characteristic that may influence a participant's functioning, this study was limited to a selection of variables that have been informed by past literature and the extant data collected. Consequently, future research may consider the addition of other characteristics and variables associated with social skills and social outcomes.

## Future Directions

Specific future directions should be considered for prospective examinations into predicting social skill improvement in PEERS to remediate possible limitations demonstrated in this study. Specifically, it is important to combat change score biases by adding increased time point analyses such as baseline and follow up measurements. By adding increased time point analyses, less biased statistical methodologies may be employed such as structural equation modeling or repeated measures analysis of variance. Not only would there be fewer statistical biases, but it would also prove interesting to examine if change scores held constant, improved, or diminished post intervention. Researchers could also consider what characteristics certain autistic individuals have that allow them to maintain or diminish intervention gains.

Future research may also consider examining different variables in their analysis. Specifically, researchers may include considerations that go beyond the heteronormative binary categorization of biological sex and gender and include more categorizations such as non-binary when examining the role that sex and gender may play in autism symptomology and social skill improvement in PEERS. Moreover, alternative variables that may be responsible for social skill challenges and predicting social skill outcomes from PEERS should be examined. For example, other cognitive abilities such as planning, working memory, mental flexibility, response initiation, response inhibition, and impulse control have been found to be lacking in some autistic individuals (73). Examples of social skills challenges due to these impairments surround isolated play, peer rejection, poor perspective taking, and issues with adaptive conversational skills. Frequently, challenges in these types of cognitive abilities and social skills are marked indicators and common challenges demonstrated by autistic individuals (74). Further examinations into these variables may provide further insight into predictors of social skill improvement in PEERS.

Finally, future research concerning predicting social skill improvement in PEERS may choose to examine other GSSIs to determine if similar results hold true when applied to different therapeutic modalities. While PEERS is considered effective, other GSSIs are commonly used and should be studied to improve both programming and to assist in the creation of novel interventions. As well, future research may attempt to replicate the findings of this study to increase the ability of generalizing the findings to real world populations. Overall, there is a large gap within the literature that proves important to further redress to improve social skill outcomes and in turn overall life outcomes for autistic individuals.

## Conclusions

Social skills are essential for positive psychosocial, academic, and employment outcomes (1, 2). However, impaired cognitive, neural, and behavioral mechanisms in many autistic individuals create a wide array of challenges reasoning, interpreting, and responding to social situations. Autistic individuals often struggle to adapt their behavior to various social environments and predict other individuals' social behaviors (75).

Fortunately, there are various GSSIs aimed at improving autistic individual's social skill functioning. Of the omnipresent GSSIs, PEERS remains highly effective in improving autistic teens social skills post intervention. Nonetheless, little is understood about characteristics that autistic teens possess and how those attributes may lead to social skill improvement in PEERS. The present study sought to examine how age, gender, EI, intellectual ability, and autism symptomatology predicted social skills outcomes of participants in PEERS to redress this relatively unknown construct. Only FSIQ and PRI had a significant effect in predicting social skills outcomes in self-report measures. These findings elucidate how certain autistic individuals may be more able to attain treatment gains in PEERS than others. Importantly, the study must be interpreted as exploratory as there are possible statistical concerns with change score analysis and other limitations. A lack of generalizability concerns is present due to possible biased self-report measures, lack of third-party reporting, and sample issues. These limitations could readily be mitigated with increased time point analysis, novel variable analysis, and replicability studies.

It would be neglectful to observe the social skill challenges faced by many autistic individuals and not seek further explorations into the specific associated mechanisms that contribute to these impairments. Although there are GSSIs specifically aimed at improving social skills, such as PEERS, it is apparent that not all participants are successful. Given the encumbrance that social skills play in an individual's psychosocial, academic, and employment outcomes, it proves important to further understand what makes autistic youth successful in improving their social competence. Consequently, continued research exploring the predictors of social skill change will allow for novel GSSIs to be created for maximal social skill improvement in various participants with heterogenous autistic symptomologies. Moving forward, it is imperative that opportunities such as positive social skill development for neurodivergent individuals is supported and better understood.

## Data Availability Statement

The original contributions presented in the study are included in the article/supplementary material, further inquiries can be directed to AM at awmccrim@ucalgary.ca.

## Ethics Statement

The studies involving human participants were reviewed and approved by Conjoint Faculties Research Ethics Board. Written informed consent to participate in this study was provided by the participants' legal guardian/next of kin.

## Author Contributions

JM gathered the data, ran the analyses, and was primarily responsible for manuscript development. AM oversaw the intervention and data collection across the various cohorts included in the study and contributed to manuscript development. Both authors contributed to the article and approved the submitted version.

## Funding

This project received funding from the Alberta Center for Child, Family, and Community Research (ACCFCR) (Grant No. 13SM-McCrimmon) for initial data included in the analyses.

## Conflict of Interest

The authors declare that the research was conducted in the absence of any commercial or financial relationships that could be construed as a potential conflict of interest.

## Publisher's Note

All claims expressed in this article are solely those of the authors and do not necessarily represent those of their affiliated organizations, or those of the publisher, the editors and the reviewers. Any product that may be evaluated in this article, or claim that may be made by its manufacturer, is not guaranteed or endorsed by the publisher.

## Footnotes

^1^The terms “autistic,” “autism” or “on the autism spectrum” will be used throughout this document rather than “with autism,” “autism spectrum disorder,” or “ASD” to respect the recommendations and practices of self-advocates (13, 14).

## References

[1] Gresham FM. Social skills assessment and intervention for children and youth. Cambridge J Educ. (2016) 46:319–32. 10.1080/0305764X.2016.1195788

[2] Wentzel KR. Peers and academic functioning at school. In: Rubin KH Bukowski WM Laursen B editors. Handbook of Peer Interactions, Relationships, Groups. New York, NY: Guilford Press (2009). p. 531–47.

[3] Miles SB Stipek D. Contemporaneous and longitudinal associations between social behavior and literacy achievement in a sample of low-income elementary school children. Child Dev. (2006) 77:103–17. 10.1111/j.1467-8624.2006.00859.x 16460528

[4] Rubin KH Coplan RJ Chen X Bowker CJ McDonald LK Heverly-Fitt S. Peer relationships. In: Bornstein MH Lamb ME editors. Developmental Science: An Advanced Textbook. 7th edn. Psychology Press (2015). p. 587–644.

[5] Dixon SD Stein MT. Understanding children: theories, concepts and insights. In: Dixon SD Stein MT editors. Encounters With Children: Pediatric Behavior and Development. 4th edn. Philadelphia, PN: Mosby (2006). p. 12–43. 10.1016/B0-32-302915-9/50006-9

[6] Soto-Icaza P Aboitiz F Billeke P. Development of social skills in children: neural and behavioral evidence for the elaboration of cognitive models. Front Neurosci. (2015) 9:333. 10.3389/fnins.2015.00333 26483621 PMC4586412

[7] Tomasello M Carpenter M Call J Behne T Moll H. Understanding and sharing intentions: the origins of cultural cognition. Behav Brain Sci. (2005) 28:675–91. 10.1017/S0140525X05000129 16262930

[8] Rubin KH Bukowski WM Bowker JC. Children in peer groups. In: Lerner RM Bornstein MH Leventhal T editors. Handbook of Child Psychology and Developmental Science. John Wiley & Sons Inc (2015). p. 175–222. 10.1002/9781118963418.childpsy40525855820

[9] Bukowski WM Laursen B Rubin KH editors. Handbook of Peer Interactions, Relationships, and Groups. 2nd ed. New York, NY: Guilford Publications (2018).

[10] Ladd GW Kochenderfer-Ladd B Eggum ND Kochel KP McConnell EM. Characterizing and comparing the friendships of anxious-solitary and unsociable preadolescents. Child Dev. (2011) 82:1434–53. 10.1111/j.1467-8624.2011.01632.x 21883155 PMC3466166

[11] Cappadocia MC Weiss JA Pepler D. Bullying experiences among children and youth with autism spectrum disorders. J Autism Dev Disord. (2012) 42:266–77. 10.1007/s10803-011-1241-x 21499672

[12] Mayes SD Gorman AA Hillwig-Garcia J Syed E. Suicide ideation and attempts in children with autism. Res Autism Spectr Disord. (2013) 7:109–19. 10.1016/j.rasd.2012.07.009

[13] Kapp SK Gillespie-Lynch K Sherman LE Hutman T. Deficit, difference, or both? Autism and neurodiversity. Dev Psychol. (2013) 49:59–71. 10.1037/a0028353 22545843

[14] Nicolaidis C Raymaker DM Ashkenazy E McDonald KE Dern S Baggs AE. “Respect the way I need to communicate with you”: healthcare experiences of adults on the autism spectrum. Autism. (2015) 19:824–31. 10.1177/1362361315576221 25882392 PMC4841263

[15] Hansen SG Carnett A Tullis CA. Defining early social communication skills: a systematic review and analysis. Adv Neurodev Disord. (2018) 2:116–28. 10.1007/s41252-018-0057-5

[16] Laugeson EA Frankel F. Social Skills for Teenagers With Developmental and Autism Spectrum Disorders: The PEERS Treatment Manual. New York, NY: Routledge/Taylor & Francis Group (2010). 10.4324/9780203867686

[17] Laugeson EA Park MN. Using a CBT approach to teach social skills to adolescents with autism spectrum disorder and other social challenges: the PEERS® method. J Ration Emot Cogn Behav Ther. (2014) 32:84–97. 10.1007/s10942-014-0181-8

[18] Laugeson EA Frankel F Gantman A Dillon AR Mogil C. Evidence-based social skills training for adolescents with autism spectrum disorders: the UCLA PEERS program. J Autism Dev Disord. (2012) 42:1025–36. 10.1007/s10803-011-1339-1 21858588

[19] Schohl KA Van Hecke AV Carson AM Dolan B Karst J Stevens S. A replication and extension of the PEERS intervention: examining effects on social skills and social anxiety in adolescents with autism spectrum disorders. J Autism Dev Disord. (2014) 44:532–45. 10.1007/s10803-013-1900-1 23893101

[20] Beauchamp MH Anderson V. SOCIAL: an integrative framework for the development of social skills. Psychol Bull. (2010) 136:39–64. 10.1037/a0017768 20063925

[21] Gresham FM Elliott SN Kettler R. Base rates of social skills acquisition, performance deficits, strengths, and problem behaviors: an analysis of the social skills improvement system- rating scales. Psychol Assess. (2010) 22:809–15. 10.1037/a0020255 20804259

[22] American Psychiatric Association. Diagnostic and Statistical Manual of Mental Health Disorders: DSM-V. 5th ed. Washington DC: APA (2013).

[23] Lord C Brugha TS Charman T Cusack J Dumas G Frazier T. Autism spectrum disorder. Nat Rev Dis Primers. (2020) 6:1–23. 10.1038/s41572-019-0138-4 31949163 PMC8900942

[24] Macintosh K Dissanayake C. Social skills and problem behaviours in school aged children with high-functioning autism and asperger's disorder. J Autism Dev Disord. (2006) 36:1065–76. 10.1007/s10803-006-0139-5 16865549

[25] McCoy A Holloway J Healy O Rispoli M Neely L. A systematic review and evaluation of video modeling, role-play and computer-based instruction as social skills interventions for children and adolescents with high-functioning autism. Rev J Autism Dev Disord. (2016) 3:48–67. 10.1007/s40489-015-0065-6

[26] Blacher J Begum G. The social and the socializing sibling: positive impact on children with autism. Except Parent. (2009) 39:56.

[27] Laws BG Feuerstein M Mason-Apps E White C. Peer acceptance of children with language and communication impairments in a mainstream primary school: associations with type of language difficulty, problem behaviours and a change in placement organization. Child Lang Teach Ther. (2012) 28:73–86. 10.1177/0265659011419234

[28] Wainscot JJ Naylor P Sutcliffe P Tantam D Williams JV. Relationships with peers and use of the school environment of mainstream secondary school pupils with asperger syndrome (high-functioning autism): a case-control study. Int J Psychol Psychol Ther. (2008) 8:25–38.

[29] Brady DI Saklofske DH Schwean VL Montgomery JM McCrimmon AW Thorne KJ. Cognitive and emotional intelligence in young adults with autism spectrum disorder without an accompanying intellectual or language disorder. Res Autism Spectr Disord. (2014) 8:1016–23. 10.1016/j.rasd.2014.05.009

[30] Bodner KE Engelhardt CR Minshew NJ Williams DL. Making inferences: comprehension of physical causality, intentionality, and emotions in discourse by high-functioning older children, adolescents, and adults with autism. J Autism Dev Disord. (2015) 45:2721–33. 10.1007/s10803-015-2436-3 25821925 PMC4555006

[31] Shamay-Tsoory SG. Recognition of ‘fortune of others' emotions in asperger syndrome and high functioning autism. J Autism Dev Disord. (2008) 38:1451–61. 10.1007/s10803-007-0515-9 18161015

[32] Hansen K Lloyd J Stough C. Emotional intelligence and clinical disorders. In: Stough C Saklofske DH Parker JDA editors. Assessing Emotional Intelligence: Theory, Research, Applications. Springer (2009). p. 219–37. 10.1007/978-0-387-88370-0_12

[33] Gross JJ. Emotion regulation: affective, cognitive, social consequences. Psychophysiology. (2002) 39:281–91. 10.1017/S0048577201393198 12212647

[34] Lopes PN Salovey P Straus R. Emotional intelligence, personality, and the perceived quality of social relationships. Pers Individ Dif. (2003) 35:641–58. 10.1016/S0191-8869(02)00242-8

[35] Kimpton CA. Social Emotional Differences of Students Who Have a Nonverbal Learning Disability or Dysphasia. (Ph.D. thesis). University of Iowa, Iowa City, United States (2011).

[36] Petti VL Voelker SL Shore DL Hayman-Abello SE. Perception of nonverbal emotion cues by children with nonverbal learning disabilities. J Dev Phys Disabil. (2003) 15:23–36. 10.1023/A:1021400203453

[37] Johnson CN Ramphal B Koe E Raudales A Goldsmith J Margolis AE. Cognitive correlates of autism spectrum disorder symptoms. Autism Res. (2021). 10.1002/aur.2577 34269525 PMC8578389

[38] Ankenman K Elgin J Sullivan K Vincent L Bernier R. Nonverbal and verbal cognitive discrepancy profiles in autism spectrum disorders: influence of age and gender. Am J Intellect Dev Disabil. (2014) 119:84–99. 10.1352/1944-7558-119.1.84 24450323

[39] Walker S Irving K Berthelsen D. Gender influences on preschool children's social problem-solving strategies. J Genet Psychol. (2002) 163:197–209. 10.1080/00221320209598677 12095089

[40] Gomes RMS Pereira AS. Influence of age and gender in acquiring social skills in portuguese preschool education. Psychology. (2014) 5:99–103. 10.4236/psych.2014.52015

[41] Hill TL Gray SA Baker CN Boggs K Carey E Johnson C. A pilot study examining the effectiveness of the PEERS program on social skills and anxiety in adolescents with autism spectrum disorder. J Dev Phys Disabil. (2017) 29:797–808. 10.1007/s10882-017-9557-x 29576723 PMC5863753

[42] Chang YC Laugeson EA Gantman A Ellingsen R Frankel F Dillon AR. Predicting treatment success in social skills training for adolescents with autism spectrum disorders: the UCLA program for the education and enrichment of relational skills. Autism. (2014) 18:467–70. 10.1177/1362361313478995 24108192

[43] McVey AJ Schiltz H Haendel A Dolan BK Willar KS Pleiss S. Brief report: does gender matter in intervention for ASD? Examining the impact of the PEERS® social skills intervention on social behavior among females with ASD. J Autism Dev Disord. (2017) 47:2282–9. 10.1007/s10803-017-3121-5 28391452 PMC6419962

[44] Wolstencroft J Robinson L Srinivasan R Kerry E Mandy W Skuse D. A systematic review of group social skills interventions, and meta-analysis of outcomes, for children with high functioning ASD. J Autism Dev Disord. (2018) 48:2293–307. 10.1007/s10803-018-3485-1 29423608 PMC5996019

[45] Lord C Rutter M DiLavore PC Risi S Gotham K Bishop S. Autism Diagnostic Observation Schedule. 2nd edn. Torrence, CA: Western Psychological Services (2012)

[46] Gresham FM Elliott SN. Social Skills Improvement System Rating Scales Manual. Minneapolis, MN: NCS Pearson (2008).

[47] Crosby JW. Test review: FM Gresham & SN Elliott social skills improvement system rating scales. Minneapolis, MN: NCS Pearson, 2008. J Psychoeduc Assess. (2011) 29:292–6. 10.1177/0734282910385806

[48] Wechsler D. Wechsler Abbreviated Scale of Intelligence–Second Edition (WASI-II). San Antonio, TX: NCS Pearson (2011). 10.1037/t15171-000

[49] McCrimmon AW Smith AD. test review: review of the Wechsler abbreviated scale of intelligence, (WASI-II). J Psychoeduc Assess. (2013) 31:337–41. 10.1177/0734282912467756

[50] Saulnier CA Klin A. Brief report: social and communication abilities and disabilities in higher functioning individuals with autism and asperger syndrome. J Autism Dev Disord. (2007) 37:788–93. 10.1007/s10803-006-0288-6 17160458

[51] Bar-On R Parker JD. The bar-on emotional quotient inventory: youth version (EQ-i:YV) technical manual. Multi Health Syst. (2000). 10.1037/t14077-000

[52] Wood LM Parker JDA Keefer KV. Assessing emotional intelligence using the emotional quotient inventory (EQ-i) and related instruments. In: Stough C Saklofske DH Parker JDA editors. Assessing Emotional Intelligence: Theory, Research, Applications. New York, NY: Springer (2009). p. 85–102. 10.1007/978-0-387-88370-0_4

[53] Constantino JN Gruber CP. Social Responsiveness Scale–Second Edition (SRS-2). Torrence, CA: Western Psychological Services (2012).

[54] Bruni TP. Test review: social responsiveness scale–Second edition (SRS-2). J Psychoeduc Assess. (2014) 32:365–9. 10.1177/073428291351752533284813

[55] Furr RM. Psychometrics: An Introduction. 2nd Edn. Thousand Oaks, CA: SAGE (2018).

[56] Jennings M Cribbie RA. Comparing pre-post change across groups: guidelines for choosing between difference scores, ANCOVA and residual change scores. J Data Sci. (2016) 14:205–30. 10.6339/JDS.201604_14(2).0002

[57] Lord FM. A paradox in the interpretation of group comparisons. Psychol Bull. (1967) 68:304. 10.1037/h0025105 6062585

[58] Bryk AS Raudenbush SW. Application of hierarchical linear models to assessing change. Psychol Bull. (1987) 101:147. 10.1037/0033-2909.101.1.147

[59] Cronbach LJ Furby L. How we should measure “change”: or should we? Psychol Bull. (1970) 74:68–80. 10.1037/h0029382

[60] Tavakol M Dennick R. Making sense of Cronbach's alpha. Int J Med Educ. (2011) 2:53–5. 10.5116/ijme.4dfb.8dfd 28029643 PMC4205511

[61] DeVellis RF. Scale Development: Theory and Applications, Vol. 26. Sage Publications (2016).

[62] Allely CS. Understanding and recognising the female phenotype of autism spectrum disorder and the “camouflage” hypothesis: a systematic PRISMA review. Adv Autism. (2019) 5:14–37. 10.1108/AIA-09-2018-0036

[63] Hiller RM Young RL Weber N. Sex differences in pre-diagnosis concerns for children later diagnosed with autism spectrum disorder. Autism. (2016) 20:75–84. 10.1177/1362361314568899 25717130

[64] Head AM McGillivray JA Stokes MA. Gender differences in emotionality and sociability in children with autism spectrum disorders. Mol Autism. (2014) 5:1–9. 10.1186/2040-2392-5-19 24576331 PMC3945617

[65] Bargiela S Steward R Mandy W. The experiences of late-diagnosed women with autism spectrum conditions: an investigation of the female autism phenotype. J Autism Dev Disord. (2016) 46:3281–94. 10.1007/s10803-016-2872-8 27457364 PMC5040731

[66] Trigueros R Sanchez-Sanchez E Mercader I Aguilar-Parra JM López-Liria R Morales-Gázquez MJ. Relationship between emotional intelligence, social skills and peer harassment. A study with high school students. Int J Environ Res Public Health. (2020) 17:4208. 10.3390/ijerph17124208 32545626 PMC7345273

[67] Sigafoos J Lancioni GE Singh NN O'Reilly MF. Intellectual disability and social skills. In: Matson JL editors. Handbook of Social Behavior and Skills in Children. Cham, Switzerland: Springer (2017). p. 249–71. 10.1007/978-3-319-64592-6_14

[68] Baron-Cohen S Golan O Chakrabarti B Belmonte MK. Social cognition and autism spectrum conditions. In: Sharp C Fonagy P Goodyer I editors. Social Cognition and Developmental Psychopathology. New York, NY: Oxford University Press (2008). p. 29–56. 10.1093/med/9780198569183.003.0002

[69] Milner V McIntosh H Colvert E Happé F. A qualitative exploration of the female experience of autism spectrum disorder (ASD). J Autism Dev Disord. (2019) 49:2389–402. 10.1007/s10803-019-03906-4 30790191 PMC6546643

[70] Jepsen MI Gray KM Taffe JR. Agreement in multi-informant assessment of behaviour and emotional problems and social functioning in adolescents with autistic and asperger's disorder. Res Autism Spectr Disord. (2012) 6:1091–8. 10.1016/j.rasd.2012.02.008

[71] Livingston LA Colvert E Bolton P Happé F. Good social skills despite poor theory of mind: exploring compensation in autism spectrum disorder. J Child Psychol Psychiatry. (2019) 60:102–10. 10.1111/jcpp.12886 29582425 PMC6334505

[72] Striano T Reid V. Social cognition at the crossroads: perspectives on understanding others. In: Striano T Reid V editors. Social Cognition: Development, Neuroscience, Autism. Hoboken, NJ: Wiley-Blackwell (2009). p. 3–16.

[73] Demetriou EA DeMayo MM Guastella AJ. Executive function in autism spectrum disorder: history, theoretical models, empirical findings, and potential as an endophenotype. Front Psychiatry. (2019) 10:753. 10.3389/fpsyt.2019.00753 31780959 PMC6859507

[74] Holmes CJ Kim-Spoon J Deater-Deckard K. Linking executive function and peer problems from early childhood through middle adolescence. J Abnorm Child Psychol. (2016) 44:31–42. 10.1007/s10802-015-0044-5 26096194 PMC4689661

[75] Happé F Frith U. Annual research review: towards a developmental neuroscience of atypical social cognition. J Child Psychol Psychiatry. (2014) 55:553–77. 10.1111/jcpp.12162 24963529

